# Variation at the major facilitator superfamily *ZIFL1* gene influences zinc concentration of barley grain

**DOI:** 10.3389/fpls.2025.1539029

**Published:** 2025-04-24

**Authors:** Girma Fana Dinsa, Joanne Russell, Brian Steffenson, Claire Halpin, Robbie Waugh

**Affiliations:** ^1^ University of Dundee, Division of Plant Sciences at the James Hutton Institute, Dundee, United Kingdom; ^2^ The James Hutton Institute, Cell and Molecular Science Department, Dundee, United Kingdom; ^3^ Department of Plant Pathology, University of Minnesota, St. Paul, MN, United States

**Keywords:** micronutrient, genome-wide association study, quantitative trait loci, zinc, barley

## Abstract

Food and nutritional security are global challenges exacerbated by an increasing human population and impacted by climate change. Barley is among the top cereal crops grown worldwide and is a strategic crop for food and nutrition security in several geographical domains. However, barley grains are generally limited in iron and zinc, two major micronutrient deficiencies affecting billions of people around the world, but particularly women and children in developing countries. One promising strategy to enhance crop micronutrient status is via biofortification, the identification and use of nutrient-rich natural variants in crop genetic improvement. Germplasm assessed as being rich in essential nutrients are used as parental materials in traditional breeding strategies. While simple in theory, directly assessing grain nutrient concentration as a phenotype in a crop breeding program is not trivial, particularly in lesser developed geographies. As an alternative, genetic diagnostics can simplify the identification of desirable progenies and accelerate the breeding process. Here we explored natural variation for grain zinc concentration within 296 Ethiopian and Eritrean barley landraces using a genome-wide association study. We found strong associations with two SNPs, both of which were located within the barley ortholog of a tonoplast-associated major facilitator superfamily (MFS) transporter gene, Zinc induced facilitator-like 1 (ZIFL1) of *Arabidopsis thaliana* (*AtZIFL1)*. Sequence-based haplotype analysis of the barley gene (*HvZIFL1)* extended this association to a 153-162 bp deletion in a non-coding region. The favourable haplotype, associated with higher grain Zn concentration, was found in ~20% of Ethiopian and Eritrean barley germplasm. Markers are designed to the diagnostic SNPs for use as molecular diagnostics in breeding for genotypes with enhanced grain Zn.

## Introduction

An estimated 3.7 billion people worldwide suffer from iron and zinc deficiencies (Karakoy et al., 2012; De Groote et al., 2021). With our general reliance on only a few crops to supply these essential micronutrients, it is considered increasingly important that we try to develop crop-specific solutions to improving nutritional quality (Stangoulis and Knez, 2022). Genetic biofortification, the natural enrichment of foods through breeding, has been suggested as a sustainable and viable approach (Stangoulis and Knez, 2022). A complementary strategy, supplementing micronutrients in fertilisers or flour (agronomic biofortification), has also been proposed (Cu et al., 2021) but is considered costly, largely ineffective and less easy to implement in subsistence farming situations. Screening germplasm to identify accessions that have increased micronutrient concentration in the grain or fruit, then understanding and incorporating the responsible beneficial alleles through marker assisted selection, is perhaps the most promising approach towards eradicating the ‘hidden hunger’ of micronutrient deficiency (Karakoy et al., 2012; Hefferon, 2019; Stangoulis and Knez, 2022; Devate et al., 2022). This global issue is particularly important in developing countries, like Ethiopia, where cereal crops such as barley are consumed as staples, despite being unable to provide sufficient critical micronutrients considered a necessary component of a healthy diet. There, 38% of children under five years of age were recently classed as stunted, a condition directly linked to low dietary zinc intake. Malnutrition and stunting are particularly prevalent in the extreme highlands where barley and faba bean are the main sources of food and nutrition. These are the principal crops grown by 4.5 million smallholder farmers with over 20 million dependent individuals. Micronutrient deficient soils are common in these regions (Karakoy et al., 2012). Indeed, it is estimated that 50% of agricultural soils in Ethiopia are zinc deficient (De Groote et al., 2021). As dietary intake is often the main source of Zn nutrition in these regions, Zn deficiency frequently reflects the widespread consumption of staple cereals that contain low zinc concentrations (Menguer et al., 2017). In barley, the endosperm and embryo generally constitute ~85-90% of the whole-grain biomass but only ~50% of the accumulated Zn, although these figures are considered genotype dependent (Menguer et al., 2017; Lombi et al., 2011).

The Consultative Group on International Agricultural Research (CGIAR) is developing biofortified crops through its *HarvestPlus* program together with an interdisciplinary alliance of research institutions, targeting incremental increases in micronutrients without compromising yield productivity or agronomic performance (Virk et al., 2021). In the context of Ethiopia, the grain Zn concentration breeding target is an increase of +10 mg/kg above a baseline of 28 mg/kg, which is based on the average grain Zn concentration of elite cultivars. The average daily *per capita* zinc requirement has been estimated as 8-11 mg for adult females and males respectively (Institute of Medicine, 2001).

As a ‘Vavilovian centre of genetic diversity’, Ethiopia has an extensive collection of barley genetic resources. The unique diversity of Ethiopian barley germplasm has long been recognised, from early studies using isozymes (Bekele, 1983) to more recent reports using gene-based SNP assays (Mamo et al., 2014; Dido et al., 2022; Yirgu et al., 2023; Caproni et al., 2023). Similarly, the range of phenotypic variation is well documented, often cited as unprecedented and unique to Ethiopia (Fantahun et al., 2023). Landraces have evolved with high levels of genetic variability, and these represent the largest component (44%) of germplasm in gene banks worldwide (van Hintum and Menting, 2003; Bockelman and Valkoun, 2010). *In situ*, these have been under continuous natural selection and human conservation for both desirable agronomic traits and phenotypic variants whose full expression is shaped by environmental influences (Mamo et al., 2014). Highly variable local landraces have been identified with large within-population diversity varying along altitudinal gradients (Hadado et al., 2009). This genetic diversity is generally determined by the type and number of genes involved, the type of trait assayed, and the influence of the environmental, amongst other factors. Several genes of small effect may be involved in expression of a quantitative trait (Yule, 2018).

Despite the importance of grain micronutrients, only one study of zinc and iron concentration in Ethiopian germplasm has been reported. Mamo et al. (2014) identified considerable variation for grain micronutrient concentration within a collection of 298 Ethiopian and Eritrean landraces that were grown in both field and glasshouse environments. Using a 9K SNP genotyping array developed for barley (Comadran et al., 2012) for a genome wide association scan (GWAS), they identified two quantitative trait loci (QTLs) on chromosome 6H associated with grain zinc concentration (Mamo et al., 2014). Here, we enhance the power of this study by increasing marker density through use of a 50K SNP array (Bayer et al., 2017). We used 296 accessions and ionomic data from field and glasshouse studies of Mamo et al. (2014) in a multi-locus GWAS model which highlighted natural variation in a *Zinc Induced Facilitator-Like 1* gene as influencing the zinc concentration of barley grain. Using a large global collection of diverse barley germplasm, we then developed a simple Kompetitive Allele Specific PCR (KASP) marker for use in marker assisted breeding.

## Materials and methods

### Phenotypic data and statistical analyses

This study was based on the Ethiopian and Eritrean Barley Collection (EEBC), a germplasm assembled by Mamo et al. (2014) from accessions held at USDA-ARS National Small Grains Collection, the International Centre for Agricultural Research in the Dry Areas (ICARDA) and N.I. Vavilov All-Russian Institute of Plant Genetic Resources (VIR). The EEBC was previously used to identify novel genes for disease resistance and other agronomic traits including grain Zn (GZC) and Fe (GFeC) concentrations. All genotypes were originally phenotyped in 2011 glasshouse and 2012 field trials in Saint Paul, Minnesota (Mamo et al., 2014; Mamo and Steffenson, 2015). The datasets were downloaded from the online archive [T3/Barley (triticeaetoolbox.org)]. Before applying any genetic analysis, missing phenotypic values (5%) were imputed using the MICE (Multivariate Imputation via Chained Equations) package in R software [CRAN - Package mice (r-project.org)] with a maximum of 50 iterations and 5 replications. MICE assumes that the missing data are Missing at Random (MAR), which means that the probability that missing data are related to observed values and can be predicted using linear regression for continuous missing values and logistic regression for categorical missing values. MICE imputes data on a variable-by-variable basis by specifying an imputation model per variable. Data were then checked for a normal distribution and descriptive statistics were used to show the spread. Assumptions of ANOVA were subsequently checked while fitting the data and estimated Best Linear Unbiased Predictor (BLUP) and Best Linear Unbiased Estimate (BLUE) models were assessed when the assumptions were violated. Biological replicates of three seeds per plot were treated as environments to estimate BLUEs and BLUPs. The BLUEs are solutions (or estimates) associated with the fixed effects and BLUPs are the solutions (identified as predictions) associated with the random effects of a model. The BLUEs, BLUPs and correlations (genetic and phenotypic) between genotypes were analysed using META-R software (Alvarado et al., 2015; 2020). Descriptive statistics such as mean, minimum, maximum, and standard deviation were obtained using the ‘*stat.desc’* function of ‘*pastecs*’ package (Grosjean et al., 2014) in the latest version of R software (R Core Team, 2020). Analysis of variance (ANOVA) was conducted using the ‘*aov’* function, available in the stats package of R software. Normality and homogeneity of variance were checked by Shapiro–Wilk (Shapiro and Wilk, 1965) and Levene’s test (Levene, 1960), respectively.

### Genetic and population structure analyses

Genetic analyses were performed on the DNA extracted from 7-10 days old leaf samples using QIAamp 96 DNA kit (Qiagen GmbH) on an automated nucleic acid purification robot. DNA quality was assessed using a Nanodrop 2000 (Thermo Fisher Scientific, Waltham, MA, United States) with a requirement for 260/280 and 260/230 ratios > 1.8. DNA was then quantified using Picogreen (Thermo Fisher Scientific, Waltham, MA, United States). 300ng lyophilised DNA per sample was sent to Geneseek (Neogen Europe, Ltd., Auchincruive, United Kingdom) for genotyping using a high throughput Barley 50K SNP array (Bayer et al., 2017) by Illumina HTS processing and HiScan array imaging (Illumina, San Diego, CA, United States). R and Theta scores were extracted from resulting *idat* files using Genome Studio Genotyping Module v2.0.2 (Illumina, San Diego, CA, United States) and allele scores were created using paRsnps (an R package for clustering, visualising, and comparing Illumina SNP genotyping data). The data were visualised with, and exported from, Flapjack, a genotype visualisation software for further analysis (Milne et al., 2010). The genotypic data were assessed for missing, heterozygous, and monomorphic markers and markers with more than 10% missing data were removed. Polymorphic markers were maintained for cluster analysis, population structure and GWAS. For cluster analysis, the R package ‘*SelectionTools’* [Index of/~software/(uni-giessen.de)] was used to read in marker data to construct Principal Components Analysis (PCA) for population structure. Euclidean distance was used to differentiate between genotypes on the PCA axes based on their genetic distance. The optimum number of clusters (K) was determined using the Elbow method which is inferred at the highest value beyond which the total within sum of squares (WSS) is no longer significantly reduced, and the gap statistic (GS) which is increasing at decreasing rate. Population structure analysis was also performed using STRUCTURE software (Pritchard et al., 2000) with a hypothetical number of sub-groups (K) ranging from 1-10 and 5000 burn-in iterations followed by Markov Chain Monte Carlo, MCMC) iterations of 5000. Five independent runs were performed for each value of K. Molecular variances (within and among population variances) were analysed in GenAlex software (Peakall and Smouse, 2012) using 10% of the markers (5,000) randomly selected from all chromosomes. The fixation index (Fst) among the EEBC panel, also known as Wright’s population differentiation statistics (Wright, 1951) was analysed in R software.

### GWAS approaches to identify associations with GZC

GWAS was initially run to compare with published results on grain colour and the QTL interval was set based on the extent of linkage disequilibrium (LD). Genome-wide and within chromosome LD (r^2^) decay were analysed in Trait Analysis by aSSociation, Evolution, and Linkage (TASSEL) software (Bradbury et al., 2007) with a sliding window of 500 markers around the significant marker to obtain the extent of inter-chromosomal LD decay. All possible candidate genes were identified based on an LD score of r^2^ ≥ 0.2 between the most significant marker associated with the trait and neighbouring SNPs. The most likely candidate genes from the list were highlighted according to their predicted functions. GWAS was performed using the grain Zn concentration data from Minnesota as described in 2.1 above and 36,431 polymorphic SNPs, and the result compared to the GWAS in the study by Mamo et al. (2014) that used 7,842 polymorphic markers. Results from several GWAS (single locus, SL_GWAS versus multi-locus, ML-GWAS) models were compared to validate the observed results. A Q-Q plot produced by the best fit model was used to determine whether the GWAS model was robust and detected only the most significantly associated SNP markers with the highest observed versus expected [-log_10_P] values. Too many markers below the Q-Q proportional line means the model is overfitting the data with false negative results, while those above the line indicate false positive results. The first three principal components (PCs) were added as covariates in the model to reasonably capture and correct for the confounding effect of population structure. GWAS was performed with the GAPIT3 package in R software (Wang and Zhang, 2021) using the BLUE data for the phenotypic trait. The results were compared between the multi-locus BLINK and the single-locus MLM models. QTL intervals were determined based on significant LD of squared correlation value r^2^ ≥ 0.20 and possible candidates were prioritised based on genes annotated with relevant functions in other species.

### Characterisation and validation of associated SNPs

Genomic sequences of the *HvZIFL1* candidate gene identified in the genome-wide association (GWA) analyses were downloaded from the genome assembly of Morex v1 (Mascher et al., 2017)(HORVU4Hr1G081570.1). Both version 2 (Monat et al., 2019) (HORVU.MOREX.r2.4HG0341120.1) and version 3 (Mascher et al., 2021) (HORVU.MOREX.r3.4H409580) were released during this study and used for subsequent genetic characterisation of the gene. The latest reference transcriptome database [Morex Gene Atlas - Hv_Mx_chr4HG27982 (hutton.ac.uk)] was used to identify potential transcripts and compared to EnsemblPlants which maintains 5 transcripts of HvZIFL1 (https://plants.ensembl.org/Hordeum_vulgare/Gene/Summary?g=HORVU.MOREX.r3.4HG0409580;r=4H:586550476-586579926;t=HORVU.MOREX.r3.4HG0409580.1;db=core). A Kompetitive Allele Specific PCR (KASP) assay was designed (**Supplementary Table 6**) to the most significantly associated marker for technology independent validation across the EEBC germplasm as well as for other barley germplasm. Haplotype analysis was performed for 194 georeferenced EEBC landrace collections using 19 SNPs from the 50K SNP platform and a locus haplotype was also plotted using 80 polymorphic SNPs across the gene obtained from exome capture data of the European Wheat and Barley Legacy for Breeding Improvement (WHEALBI) project (Russell et al., 2016; Bustos-Korts et al., 2019).

Amino acid sequences of ZIFL1 paralogues were downloaded from *EnsemblPlants* and BLAST searched in the National Centre of Biotechnology institute (NCBI) database. The top 100 sequences were downloaded and used for multiple alignment in Jalview 2.11.2.1 software (Waterhouse et al., 2009) and sequences were uploaded in the *Multiple Expectation maximisation for Motif Elicitation (MEME)* online database [ https://memesuite.org/meme/tools/meme] to explore protein motifs and hence possible similar functions (Bailey and Elkan, 1994). Known conserved motifs and new signatures were discovered using detailed analysis in the latest version of Jalview (Waterhouse et al., 2009) and by multiple alignment using Muscle (Edgar, 2004).

## Results

### Marker dense GWAS directly reveals a highly plausible candidate gene associated with grain zinc concentration

**Figure 1:**
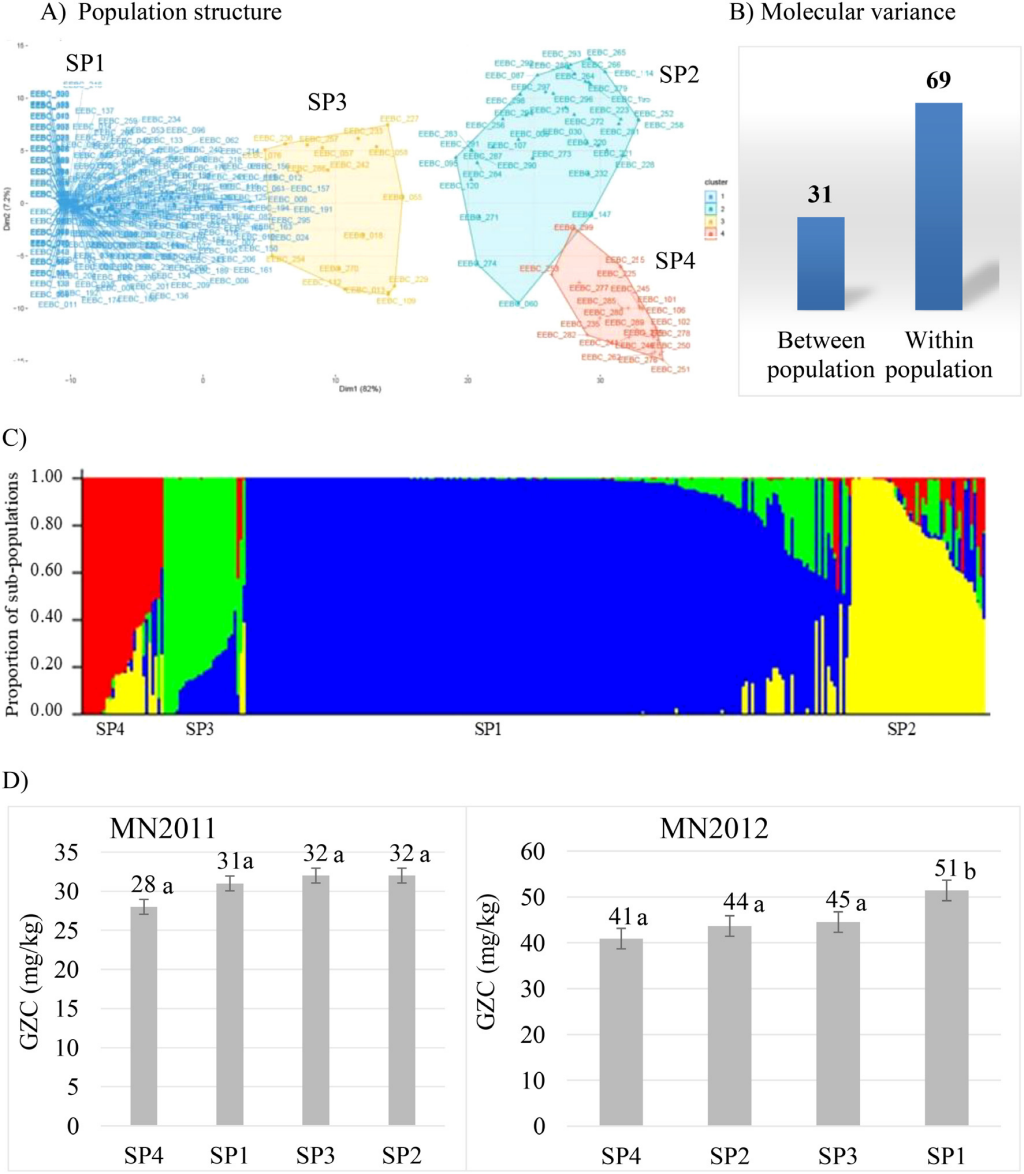
Population structure of the EEBC panel: **(A)** showing four clusters using PCA; **(B)** molecular variance percentage within and between populations; **(C)** population structure and admixture according to the output from STRUCTURE software (SP1 is the sub-population representing 72% of the EEBC; SP2 represents 14.2%, SP3 7.4% and SP4 6.4%); **(D)** average GZC (Grain Zinc Concentration, mg/kg) of sub-populations at two test locations (MN2012, Minnesota 2012 field trial; MN2011, Minnesota 2011 glasshouse trial). Figures with the same letter are not statistically significantly different.

Population structure is one of the confounding factors limiting efficiency of detecting quantitative trait loci in genome-wide association studies. It is hence crucial to characterise and determine the diversity and genetic structure existing within the germplasm panel. To this end, we used the same extensive EEBC panel assembled by University of Minnesota (Mamo et al., 2014) to determine the level of population structure. We genotyped the accessions using the barley 50K SNP assay (Bayer et al., 2017) and after filtering to remove failed, missing (>10% removed), monomorphic and low frequency (< 5%) markers, 36431 polymorphic gene-based and physically mapped SNPs remained. We observed four major clusters of the EEBC germplasm with 72% present in subpopulation 1 (SP1) with no partitioning by row type or by geography (**Figures 1A, C**). Molecular variance explained 69% and 31% (**Figure 1B**) of genetic diversity within and among subpopulations of the EEBC, and the first two principal components (PC1 and PC2) explained 82% and 7% of the genetic variation between the sub-populations, respectively. To examine diversity of the EEBC in a global context 50K SNP data was combined with the 1000 accession core barley collection in the German Federal Gene bank IPK Gatersleben which had been genotyped using the same platform (Darrier et al., 2019). Principal Component Analysis (PCA) uncovered the level of diversity and population structure with the first (PC1) and second (PC2) axes explaining 41% and 25% of the variation, respectively identifying four separate genotypic clusters (**Supplementary Figure 1A**). The Ethiopian (EEBC) germplasm was shown to form tight clusters with 238 lines (80%) of the 296 accessions forming a single main cluster (highlighted in green in **Supplementary Figure 1A**). Although 46 Ethiopian lines were among the core panel, only half grouped within the EEBC cluster. Molecular variance depicted 57% of the variation within and 43% between sub-populations (**Supplementary Figure 1B**).

The phenotypic data (Mamo et al., 2014) was analysed and descriptive statistics used to summarise grain Zn concentration of the EEBC panel in both Minnesota glasshouse and field trials (**Supplementary Table 1**). Grain Zn concentration ranged between 6-72 mg/kg in the glasshouse and 20-87 mg/kg in the field. Analysis of variance (ANOVA) depicted significant difference between genotypes (G), locations/environment (E) and genotype-by-location/environment interaction [GEI] (**Supplementary Table 2**). Genotype, location/environment (E) and genotype-by-environment interaction (GEI) contributed 32%, 36%, and 21% to the total variance (% total sum of squares, %TSS) (**Supplementary Table 2**). In contrast, within-population analysis revealed no statistical difference among the sub-populations (**Figure 1D**) in the 2011 glasshouse trial with a mean GZC ranging from 28 mg/kg for SP4 to 32 mg/kg for SP2. The 2012 field trial, however, depicted significantly higher GZC of 51 mg/kg for the SP1 compared to the lowest grain Zn concentration of 41 mg/kg for SP4. The two sub-populations SP2 and SP3 had statistically similar grain Zn concentration of 44 mg/kg and 45 mg/kg, respectively. The SP4 is comprised of 20 two-rowed elite cultivars or breeding lines including the UK standard check variety known as Concerto.

**Figure 2:**
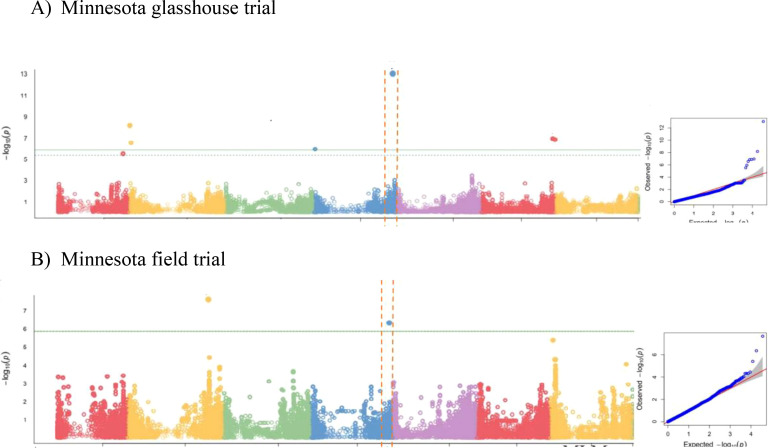
GWAS results using BLINK model for grain Zn concentration of: **(A)** 2011 glasshouse trial at Saint Paul, MN; **(B)** 2012 field trial at Saint Paul, MN.

We then performed GWAS, accounting for the observed population structure, and compared several single locus (SL-GWAS) and multi-locus (ML-GWAS) models with the objective of identifying consistent associations regardless of the statistical model. GWAS for grain zinc concentration (BLUE data, Mamo et al., 2014) from glasshouse and field trial was analysed using three different statistical models (**Figures 2A, B**; **Supplementary Table 4**; **Supplementary Figures 2A, B**). For the glasshouse data GWAS consistently revealed a significant association at the bottom of chromosome 4HL. From the field data, only BLINK detected a significant association at this locus (**Supplementary Figure 2B**). Given grain Zn concentration is likely to be under complex genetic control, on weight of evidence we decided to investigate the terminal 4H region in more detail. High SNP marker density allowed the association boundaries to be defined accurately based on the drop in LD (**Supplementary Figure 3**) between the most highly associated and physically adjacent non-significant markers. The most highly associated SNP (JHI-Hv50-2016-265280) was located within a gene annotated as a member of the Major Facilitator Superfamily (MFS) of transporters that includes Zinc Induced Facilitator (ZIF) and ZIF-Like (ZIFL) proteins. As no other annotated genes within the region appeared strong candidates, we prioritised HvZIFL1 as a strong causal gene candidate for more detailed analyses.

**Figure 3:**
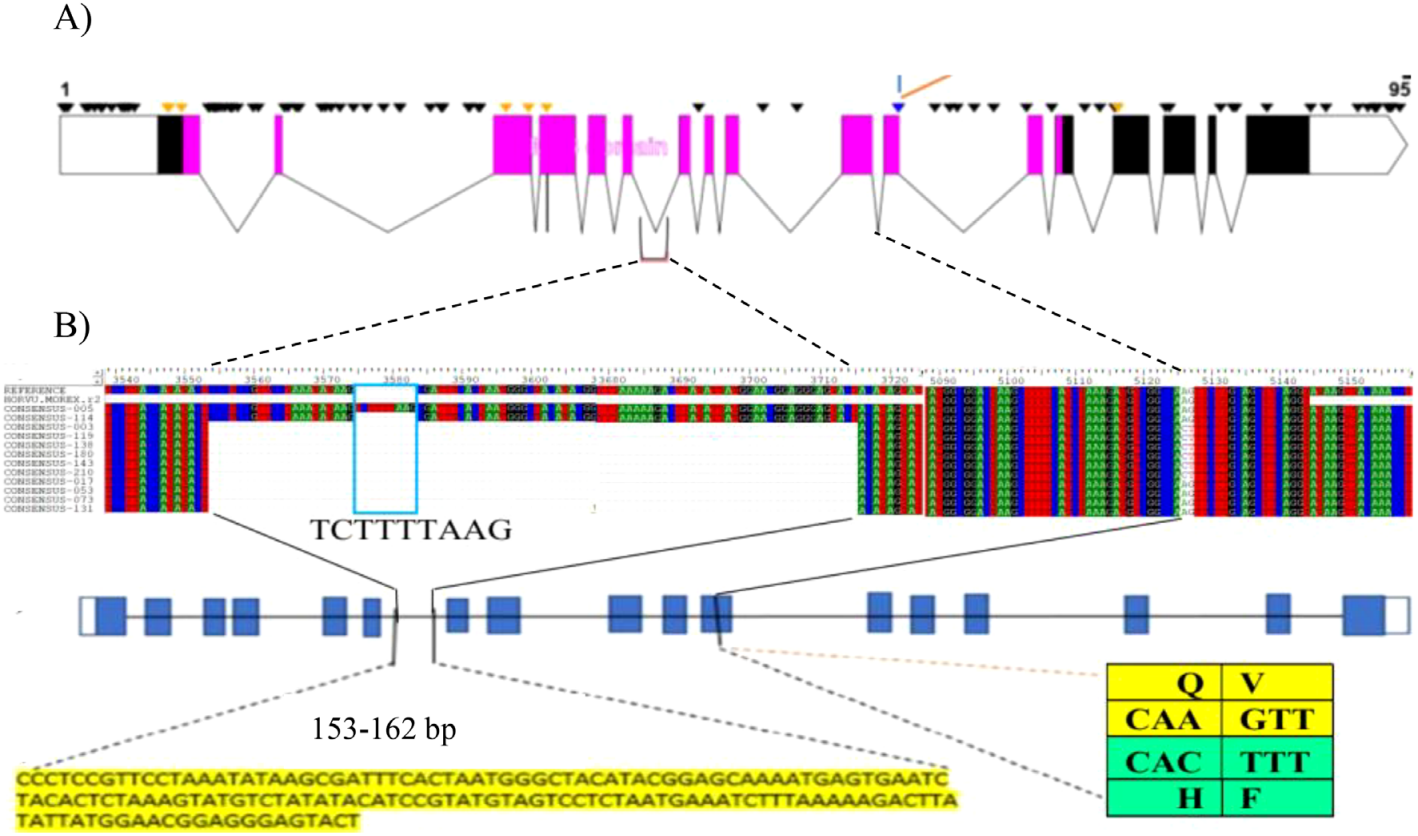
**(A)** HvZIFL1 gene model (based on Morex v1 assembly) showing 95 polymorphic sites identified using Sanger sequencing. The diagnostic marker from the 50K SNP array and an adjacent SNP identified by sequencing are parts of two codons embeded in exon-11(indicated by branching lines). The polymorphic sites include 153-162 bp INsertion DELetion (INDEL) between exon-6 and exon-7 and the diagnostic marker (Hv50k-2016-265280) annotated on 50K SNP array and an adjacent SNP identified by sequencing. The pink boxes indicate exons in the MFS domain. **(B)** multiple sequence alignment of HvZIFL1 among 12 EEBC lines (top section). Gene model with blue blocks indicate exons interspaced with lines indicating introns and polymorphisms positions (middle section). The two important polymorphism sites are indicated with 153-162 bp INDEL sequence and the SNP changes at exon-11 with codons indicated in the table (bottom section). The last SNP of the first codon (CAA) is the wild type allele coding for the amino acid Glutamine (Q) while the mutation (CAC) causes amino acid change to Histidine (H). similarly, the first SNP of the second codon (GTT) is the wild type allele coding for Valine (V) while the mutation changes to Phenylalaine (F).

We extracted the complete *HvZIFL1* (HORVU4Hr1G081570.1) gene sequence from Morex reference genome assembly version 1 (Mascher et al., 2017) to investigate molecular polymorphism, and again when version 2 became available (Monat et al., 2019). The protein encoded by HvZIFL1 consists of 493 amino acid residues and its secondary structure was predicted to contain a typical MFS protein structure with 12 membrane spanning domains known as transmembrane helices (TMH) that facilitate substrate transport across the biological membrane (**Supplementary Figure 4**). We predicted that a mutation changing Glutamine to Histidine (Q330H) and an adjacent Valine to Phenylalanine (V331F) respectively) located at the gate of TMH8 (**Figure 3B**; **Supplementary Figure 4**) may be functionally disruptive (positive mutation because it increase grain GZC). Further examination of the 50K SNP data revealed that 19 polymorphic SNPs were located within *HvZIFL1* and haplotype analysis revealed eight haplotypes (**Table 1**) across the 297 (296 EEBC plus *Concerto*) accessions with frequencies ranging from 0.007 (2 accessions) to 0.581 (172 accessions). In the combined EEBC and IPK dataset (Darrier et al., 2019), we observed 10 unique haplotypes with frequencies ranging from 0.003 to 0.536. The most significantly associated GWAS SNP was a T to G transversion that was unique to Haplotype 1 (**Tables 1**, **2**) which had significantly higher grain zinc concentration (47.6 mg/kg) when compared to all other haplotypes (42.8 mg/kg for haplotype 5 to 34.1 mg/kg for haplotype 4).

**Table 1:** Sequence of haplotypes around the diagnostic marker of the EEBC accessions.

Haplotype	EEBC Frequency	EEBC + IPK frequency	Sequence
Haplotype 1	0.199	0.181	GAGGTTAAAGGTTGAGGCC
Haplotype 2	0.034	0.003	AGGTGAGGATGCCGAACCT
Haplotype 3	0.017	0.003	AAGGTAGAATGTCGGAGCT
Haplotype 4	0.128	0.163	AGATGAGGGTACCGGACTT
Haplotype 5	0.010	0.038	AGATGAGGATACCGGACTT
Haplotype 6	0.581	0.536	GAGGTTAAATGTTGAGGCC
Haplotype 7	0.007	0.038	GAGGTTAAATGTTTAGGCC
Haplotype 8	0.024	0.012	GAGGTTGAATGTTGAGGCC
Haplotype 9	–	0.015	AGGTGAGGATGCCGGACCT
Haplotype 10	–	0.012	AGGTGAGGGTACCGAACTT

The EEBC panel contained 297 accessions (296 EEBC lines + the standard line Concerto) and the IPK panel contained 46 collections from Ethiopia (Darrier et al., 2019).

**Table 2:** Frequency of associated SNP across barley germplasm collections.

Germplasm	Number of lines	JHI-Hv50k-2016-265280	
		G*	T
Cultivar	575	0.047	0.953
Landraces	197	0.046	0.954
Wild barley	258	0.004	0.996
EEBC	292	0.202	0.798

*Denotes diagnostic SNP for high grain zinc concentration on the forward strand (C on the reverse strand).

### Ethiopian and Eritrean barley germplasm possess unique variation in *HvZIFL1*

**Figure 4:**
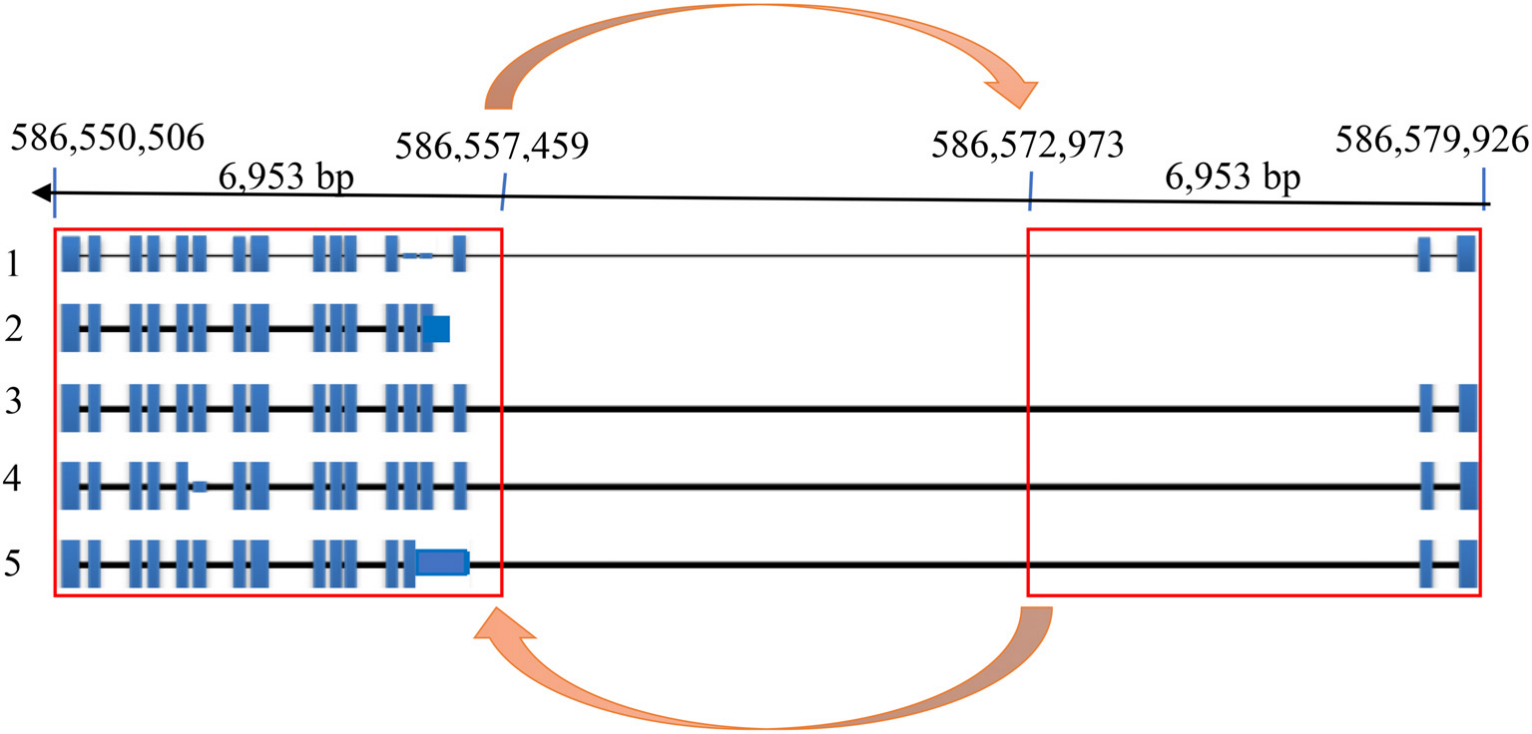
HvZIFL1 gene model on the reverse strand of Morex genome assembly v3 (Mascher et al., 2021) showing 5 alternative transcripts. The orange arrows indicate 6,953 base pairs of duplicated HvZIFL1 gene section with a 15,514 bp flanking intronic region.

We designed 12 pairs of primers (**Supplementary Table 5**) to amplify the complete *HvZIFL1* based on Morex version 1 and PCR-sequenced the gene in twelve genotypes that represented high and low grain Zn concentration. A total of 95 SNP polymorphisms were identified, almost all of which are silent/synonymous changes occurring within non-coding regions (black inverted triangle marks, **Figure 3A**). The two exceptions were the diagnostic SNP marker JHI-Hv50k-2016-265280 identified by GWAS, and an adjacent SNP discovered by Sanger sequencing using primer pair 9 (**Supplementary Table 5**; indicated with branching lines in **Figure 3**). Sequencing and alignment also revealed a variable INDEL of either 162 bp or 153 bp in length within the non-coding region between exons six and seven (**Figure 3B**). This INDEL was confirmed using agarose gel electrophoresis and was present in 10 of the 12 Ethiopian barley sequences, with EEBC_005 having a 162 bp INDEL and EEBC_114 the same 153bp INDEL as the Morex reference annotation (**Figure 3B**). Assaying the INDEL across the EEBC identified 51 lines with the 153bp insertion identical to the reference cultivar Morex (**Supplementary Table 8**). The highly significant markers from GWAS were found in Exon 11 changing a CAA (Glutamine, Q) to CAC (Histidine, H) with the adjacent mutation changing GTT (Valine, V) to TTT (Phenylalanine, F). Somewhat surprisingly, the most significantly associated GWAS SNP was not in complete LD with the 153 bp deletion. Further investigation of the HvZIFL1 gene sequence revealed duplication of ~7 kilobase pairs with 15.5 kbp interspaced sequence (**Figure 4**).

**Figure 5:**
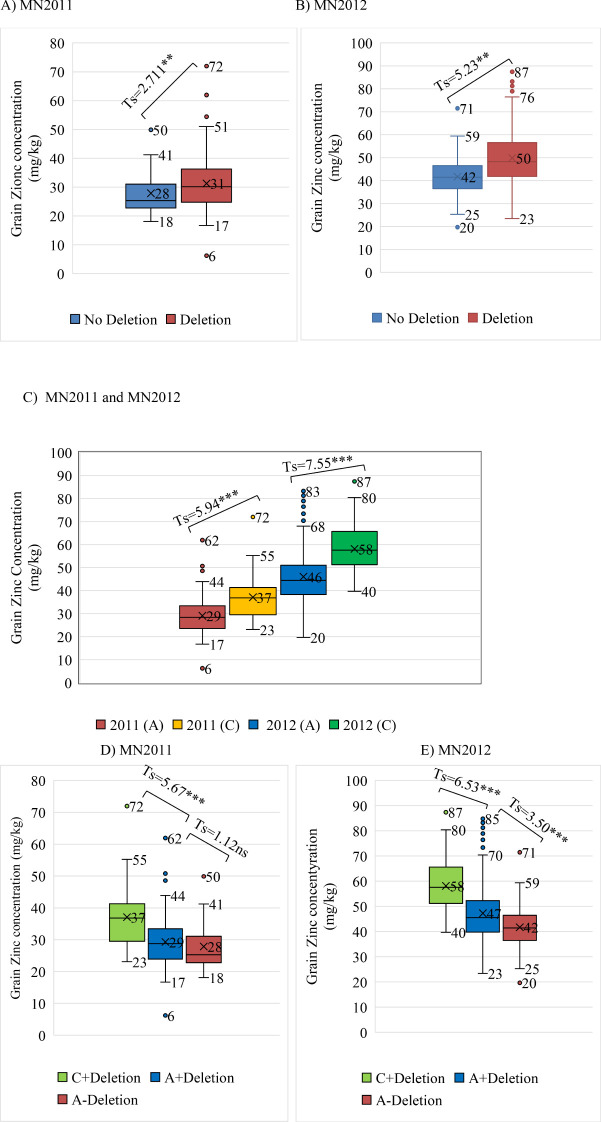
Box plots and t-tests of grain zinc concentration associated with the diagnostic SNP marker and the INDEL (153-162 bp). **(A, B)** The INDEL effect on GZC of glasshouse and field trials at Minnesota in 2011 and 2012, respectively; **(C)** Marker effect on GZC of glasshouse and field trials at Minnesota; **(D, E)** Combined INDEL + marker effects on GZC of glasshouse and field trials at Minnesota in 2011 and 2012, respectively.

Because the 50K SNP assay has been widely used by the barley research community as a platform for genetic analysis and marker assisted selection (MAS), we were able to examine the distribution of the highly associated marker JHI-Hv50k-2016-265280 across a wide range of germplasm including 575 cultivars, 197 landraces and 258 wild barley accessions (**Table 2**). The frequency of the minor allele associated with increased grain zinc is 0.202 in the EEBC compared to 0.047 in cultivars and 0.004 in wild barley accessions. These frequencies observed in both the landrace and wild germplasm are below 0.05 which is usually chosen as the threshold for robust marker:trait detection in association studies and would therefore not have been significant in any of these germplasm sets. We also assembled haplotype sequences from the genetically diverse WHEALBI collection (N= 432) (Bustos-Korts et al., 2019) using 80 polymorphic SNPs discovered in exome capture data. We identified 38 unique haplotypes where the favourable JHI-Hv50k-2016-265280 ‘C’ allele was found only in Haplotype 18 (Hap_18), comprised of 40 lines of which 30% were from Ethiopia and Eritrea (**Supplementary Tables 7**, **9**, **10**). Analysis of grain Zn concentration showed a significant effect associated with both the 153bp INDEL and the diagnostic marker, individually and in combination (**Figures 5A–E**). The boxplots and t-tests showed that genotypes with the 153 bp deletion in *ZIFL1* had a mean grain Zn concentration of 31 mg/kg compared to 28 mg/kg for those without the deletion (t=2.71**, **Figure 5A**) using the Minnesota (glasshouse) data. The difference between these groups in the field was 8 mg/kg (t= 5.23**, **Figure 5B**). The JHI-Hv50k-2016-265280 SNP effect for the glasshouse and the field trials showed an 8 mg/kg (t= 5.94***) and 12 mg/kg (t= 7.55***) difference between alleles (**Figure 5C**). Variability in grain Zn concentration was also apparent in the data across the two locations/years (**Figures 5D, E**). In the 2011 glasshouse trial, deletion had an average effect of 1 mg/kg (t-statistics = 1.12 ns) while the SNP marker had a mean effect of 8 mg/kg (t=5.67***, **Figure 5D**). In the 2012 field trial, deletion had an average effect of 5 mg/kg (t= 3.50***) while the SNP effect was 11 mg/kg (t= 6.53***). Interestingly, the combined effect of the 153bp INDEL and the favourable allele increased grain Zn concentration level by 9 and 16 mg/kg in the glasshouse and field conditions respectively in Minnesota.

### HvZIFL1 on 4H is the only one of the 34 annotated ZIFL genes associated with grain zinc concentration

A BLAST search of the amino acid sequence of the candidate HvZIFL1 gene identified 33 paralogues distributed across the barley genome (**Supplementary Table 3**). For most of these genes, SNPs were present on the 50K chip and almost all were polymorphic with minimum allele frequency (MAF) values ranging from 0.021 to 0.476. However, none of the SNPs were associated with grain zinc concentration except the HvZIFL1 on 4H with an MAF of 0.202. We then compared the HvZIFL1 protein sequence with its cereal orthologs and identified 30 short conserved sequence motifs that may be related to both conserved and unique functions of the protein. The motifs could be categorised into three major and three minor groups (**Supplementary Figure 5**). The first major group (G-I) contained HvZIFL1 and 10 other ZIFL genes and exhibited 11 conserved motifs. The second major group (G-II) consisted of 9 ZIFL proteins with 10 unique motifs while the third group (G-III) contained 9 ZIFL genes with 12 unique sequence motifs including motif 6 and 15 which were conserved in all groups. The three minor groups consisted of 1-2 ZIFL genes with four conserved motifs.

### HvZIFL1 gene expression supports a role in micronutrient uptake

We used the cv. Morex EoRNA gene expression atlas to explore the temporal and spatial transcript abundance and post transcriptional variation of *HvZIFL1* (https://ics.hutton.ac.uk/morexgeneatlas/index.html). HvZIFL1 (transcript Hv_Mx_chr4HG27982 in MorexGeneAtlas) (Guo et al., 2025) was detected as multiple alternative transcripts (**Supplementary Figure 6**) with all, including the most abundant, expressed exclusively in root tissues with no indication of expression in any above ground tissues, including the developing grain (**Supplementary Figure 7**). These observations are consistent with HvZIFL1 contributing to variation in total Zn uptake from soil. Whether subsequent transport to the developing grain is passive (e.g. via the transpiration stream) or active (involving other Zn transporters) could not be determined but does not apparently involve HvZIFL1. Furthermore, there was little variation in the level of *HvZIFL1* expression in roots when Zn (or other heavy metals) were added to the growth medium suggesting its expression is not responsive to Zn availability (Kintlová et al., 2017) but rather is genetically pre-determined in different genotypes.

### Development of a Kompetitive Allele Specific PCR assays for breeder’s toolbox

The diagnostic SNP marker (JHI-Hv50k-2016-265280) identified by GWAS was selected for validation as a tool for marker-assisted selection (MAS) using a KASP assay. Alternative alleles in all EEBC accessions were then classified using the KASP assay. 237 lines had the JHI-Hv50k-2016-265280 ‘A’ allele associated with lower grain zinc concentration and 59 lines the JHI-Hv50k-2016-265280 ‘C’ allele associated with higher grain zinc concentration (**Figures 5A, B**). Comparing the data from the KASP assay and the 153bp deletion PCR-based size marker, the JHI-Hv50k-2016-265280 ‘C’ allele was only associated with the deletion whereas the ‘A’ allele was associated with and without the deletion in a frequency of 0.212 (‘A’ allele, no deletion) and 0.787 (‘A’ allele, with deletion). The impact of using both markers increased the differences of grain zinc concentration to 11 mg/kg and 16 mg/kg for glasshouse and field data, respectively (**Figures 5C, D**).

## Discussion

Since its domestication over 10,000 years ago, barley landraces have been cultivated widely as a dependable crop in areas with diverse and extreme environmental conditions (Grando and Macpherson, 2005). These landraces have evolved across a wide geographical range and exhibit high genetic variability. Today, due largely to their geographical and morphological diversity, landraces comprise the largest proportion of germplasm (44%) in worldwide gene banks (van Hintum and Menting, 2003; Bockelman and Valkoun, 2010). In Ethiopia, Hadado et al. (2010) confirmed the high variability of local populations of barley landraces with large within-population diversity varying along an altitudinal gradient. This rich phenotypic diversity (Mamo et al., 2014) is the result of variation in both major genes and quantitative characters comprised of multiple genes of small effect (Yule, 2018). In a recent gene bank genomics study based on extensive Genotyping by Sequencing (GBS) data, Milner et al. (2019) observed that 5000 Ethiopian lines out of > 20,000 geographically diverse accessions held by the German Federal Gene bank at IPK-Gatersleben formed a distinct and unique genetic cluster. Using the barley 50K SNP array (Bayer et al., 2017), Darrier et al. (2019) genotyped 1,000 representatives of the core of this collection including 46 accessions from Ethiopia. The SNP data reflected the observations using GBS, with a similar outcome reported using exome capture data (Russell et al., 2016; Chen et al., 2022). Here, using the 50K SNP platform we genotypically and phenotypically characterised 296 Ethiopian and Eritrean genotypes (the EEBC panel) to identify associations between molecular markers and phenotypic traits. The EEBC is somewhat unique as it is assembled from germplasm that has experienced an extended period of geographic isolation from the rest of the global barley population. Our genotypic data again show that the EEBC accessions are genetically closer to each other than to other global barley accessions (Milner et al., 2019). Genotype By Sequencing (GBS) data revealed three major clusters (K=3) in the world barley collection (Milner et al., 2019) described as two-row spring type, six-row mixed type, and Asian six-row types (Darrier et al., 2019). Our pooled analysis of the two datasets combined was enabled by use of the 50K SNP array and identified four clusters, with EEBC forming two tightly clustered groups (Van Leur and Gebre, 2003) emphasizing the uniqueness of the Ethiopian barley germplasm, and thus its potential as a source of potentially valuable alleles for trait improvement.

One such trait could be biofortification, and biofortification of food crops with enhanced mineral nutrient concentration has become a research priority as a means to combat micronutrient deficiencies in human diets (Cu et al., 2021). One strategy is to identify genetic markers associated with high grain nutrient concentration and then use marker assisted breeding to develop nutrient-rich varieties (Karakoy et al., 2012; Hefferon, 2019). Identifying linked genetic markers and genes using GWAS has been successful for grain Zn concentration in rice (Cu et al., 2021) and wheat (Velu et al., 2018), Na in barley (Houston et al., 2020), and other nutrients in wheat (Alomari et al., 2021). Here we focused our analyses on Zinc given it remains the most widespread micronutrient deficiency in agricultural lands around the world, causing significant yield reduction and loss of nutritional quality (Welch and Graham, 2004). Indeed, cereal-based diets with low Zn concentration are considered a major reason for widespread human Zn deficiency, especially in developing countries (Biesalski, 2013).

Cereal species and genotypes vary widely in their grain Zn concentration, and this is affected by both genetic and environmental factors. For example, soil pH affects nutrient availability for plant growth. A soil pH of between 5.5 and 6.5 is optimal for Zn availability for plant uptake and in acidic soils, increasing soil pH from 5.2 to 6.8 by liming resulted in an approximate 10-fold decrease in plant Zn concentration (Parker and Walker, 1986). In addition, Zinc availability is often reported to be low in soils with high organic matter content due to increased adsorption of Zn by organic ligands and components. Low soil moisture and low temperature also contribute to Zn deficiency (Moraghan and Mascagni, 1991). However, the physiological and molecular mechanisms of Zn deficiency tolerance are only just beginning to be understood (Hacisalihoglu and Kochian, 2003). The importance of transporters is reflected in reports that suggest about 7% of *E. coli* proteome, 2.5% of that of *Arabidopsis thaliana*, and 7% of the predicted genes in *Vitis vinifera*, are transporter proteins (Patil et al., 2019). Zn-efficient genotypes may produce high amounts of chelators that bind Zn in the rhizosphere and increase its physiological availability, while Zn transporters in the root help sustain Zn uptake during periods of Zn deficiency. Consequently, grain Zn concentration levels are influenced by genotype, environment and genotype-by-environment interactions (GEI).

Based on the data from two locations in Minnesota, these factors respectively contributed 32%, 36% and 21% of the total variation of GZC in the EEBC panel. Different levels of baseline grain Zn concentration have been established for commercial varieties of staple crops growing in different regions ranging from 16 mg/kg for rice to 25 mg/kg in maize and wheat with their corresponding breeding targets for improvement (Virk et al., 2021). The barley baseline grain Zn concentration in the EEBC has been set here to an average grain Zn concentration of 28 mg/kg and the breeding target of +10 mg/kg increase to develop elite barley varieties that meet the average daily human Zn requirement of 10 mg. Descriptive statistics showed within population difference in grain Zn concentration for the Minnesota 2012 field data but not for the 2011 glasshouse trial. For example, the average grain Zn concentration was significantly higher for SP1 (51 mg/kg) compared to the other three EEBC sub-populations.

Here we used GWAS, incorporating different statistical models to robustly map the association of phenotypic grain Zn concentration data in the EEBC to a specific location on barley chromosome 4H. We then used reference genomic information from the cultivar Morex (Mascher et al., 2017; 2021; Monat et al., 2019) to identify natural gene-level variations in *HvZIFL*1 arguing that this gene is likely involved in Zn homeostasis in the EEBC germplasm collection. Sequence characterisation revealed two important types of polymorphism: intronic deletions and point mutations. The majority of the latter were silent mutations. The INDEL and the highly associated SNP marker identified by GWAS together differentiated the EEBC germplasm into 20% high and 80% low Zn haplotypes. We also observed that the significance of the genetic association with these markers was environmentally sensitive. However, despite this, the combined effect of both SNP and INDEL markers resulted in an increase of 9 and 16 mg/kg in the glasshouse and field conditions at Minnesota, respectively.

Zn is a key structural component of many plant proteins including transcription factors and metalloenzymes (Figueiredo et al., 2012). Lira-Morales et al. (2019) reviewed several families of proteins implicated in Zn homeostasis in barley which include metal tolerance proteins (MTPs) that are involved in vacuolar Zn storage (Desbrosses-Fonrouge et al., 2005); heavy-metal ATPases (HMAs), which are involved in heavy metal tolerance and Zn storage in the vacuole (Morel et al., 2009); Natural Resistance Associated Macrophage Protein 4 (NRAMP4) which is involved in the root to shoot Zn translocation (Verret et al., 2004); the yellow stripe-like (YSL) family that are associated with Zn transport to reproductive tissues and leaves (Waters et al., 2006); and Plant Cadmium Resistance 2 (PCR2) which is associated with long-distance Zn transport (Song et al., 2010). Of particular relevance to the work we describe here, Haydon and Cobbett (2007) described a Zinc Induced Facilitator (ZIF1) gene in *Arabidopsis thaliana* along with two ZIF1-like (ZIFL1 and ZIFL2) genes. A detailed analysis of the ZIFL family of plant genes was given by Ricachenevsky et al. (2011).

Transport of Zn from the root to the shoot and its translocation within the plant has been reported to involve proteins belonging to different families (Sharma et al., 2019). ZIFL genes are members of the largest group of secondary active transporters (carrier proteins) that transport solutes against concentration gradients using active energy sources (Patil et al., 2019). They are members of the Major Facilitator Superfamily (MFS) of proteins that transport a broad range of molecules through three distinct kinetic mechanisms: uniport, antiport or symport. ZIFL family proteins described in *Arabidopsis thaliana* were expressed in the tonoplast (vacuolar membrane) and implicated in providing tolerance to excess Zn (Haydon and Cobbett, 2007). Our BLAST searches identified 33 paralogs of HvZIFL1 distributed across the barley genome and 345 homologous genes (or gene fragments) in other species including 13 in rice (Ricachenevsky et al., 2011) and 35 in wheat (Sharma et al., 2019). All belong to the MFS protein family where crystal structures suggest a distinctive protein constitution consisting of two similar (N-terminus and C-terminus) domains each containing multiple transmembrane-spanning α-helices (TMH domains) (Niño-González et al., 2019), along with an MFS_1 domain (Pfam ID: PF07690) and an antiporter domain (Meena et al., 2022). The latter surround a substrate translocation pore that operates via a rocker-switch movement of the two halves of the protein (Law et al., 2008). MFS proteins differentiate between a wide range of substrates and while we suggest *HvZIFL1* is involved in Zn uptake, the functions of all 33 HvZIFL1 paralogs distributed across the barley genome are not yet elucidated. There is evidence in Arabidopsis that AtZIFL2 is involved in Potassium and Cesium homeostasis (Remy et al., 2015).

In summary, by applying a GWAS on the EEBC we identified highly significant genetic associations between barley grain Zn concentration and SNPs embedded in *HvZIFL1* on the long arm of chromosome 4H. *HvZIFL1* is exclusively expressed in barley root tissues, supporting its role in Zn uptake from the environment, and appears to be unaffected by the concentration of Zn or other tested heavy metals in the growth medium (Kintlová et al., 2017). Using barley reference and pan-genome assemblies (Jayakodi et al., 2020), we characterised DNA sequence polymorphisms in *HvZIFL1* and found that an intronic deletion of 153 bp along with two adjacent point mutations in exon 11 were associated with variation in grain zinc concentration. We found that the favourable haplotype was present in approximately 20% of Ethiopian and Eritrean barley germplasm and the two types of polymorphism together were associated with higher grain Zn accumulation. As is common for multigenic traits, *HvZIFL1* has a variable effect size in different growing environments. In a world barley collection, variant *HvZIFL1* types were mainly found in East African germplasm while it is largely genetically fixed in global landraces and wild barley accessions. The unique and specific distribution of the desirable variant of *HvZIFL1* clearly illustrates the value of using different gene pools for GWAS as the observed associations would not have been found in germplasm from outside of Ethiopia and Eritrea. We designed KASP markers to the polymorphic SNPs and validated them for potential use in marker-assisted selection in breeding programs inside and outside of their regional origin. At the genomic level, we identified 33 ZIFL paralogs distributed within the barley genome whose functions are not yet elucidated. Based on the function of characterised ZIFL proteins it seems likely that they will be redundantly involved in functions associated with micronutrient transport from the environment to the root, from the root to the shoot and from the shoot to the grain. Their full functional characterisation remains largely to be described.

In conclusion, eliminating both overt and hidden hunger is top of the global food and nutrition security agenda. Zn deficiency affects one-third of the global population and is most widespread in regions with Zn-deficient soils. By exploring naturally occurring variation in micronutrient concentration, we took a genetic approach to develop genetic markers associated with grain Zinc concentration. We, therefore, propose the combined use of the simple deletion and mutation genetic markers we detected here, to be used as a fast-track approach to barley breeding for enhanced Zn concentration. This could significantly improve Zn nutrition of smallholder farmers in certain geographies where barley is a daily staple.

## Data Availability

The raw data supporting the conclusions of this article will be made available by the authors, without undue reservation.
