# Supplementary material for: Variation at the major facilitator superfamily ZIFL1 gene influences zinc concentration of barley grain
Source: Front Plant Sci. 2025 Apr 24;16:1539029. doi: 10.3389/fpls.2025.1539029 (PMC12058730; doi:10.3389/fpls.2025.1539029)
Supplement: Supplementary file 1 [file DataSheet1.docx]

Supplementary Material

# Supplementary Tables

**Supplementary Table 1.** Descriptive statistics of grain Zn concentrations (GZC, mg/kg) for Minnesota 2011 glasshouse and 2012 field trials.

| Statistic | MN-2011 | MN-2012 |
| --- | --- | --- |
| Mean | 31 | 47 |
| Standard error (SE) | 0.5 | 0.67 |
| Median | 30 | 45 |
| Mode | 37 | 37 |
| Standard deviation (SD) | 8.59 | 11.61 |
| Sample variance | 74 | 135 |
| Kurtosis | 2.3 | 0.8 |
| Skewness | 0.99 | 0.86 |
| Range | 66 | 67 |
| Minimum | 6 | 20 |
| Maximum | 72 | 87 |

MN-2011= Minnesota glasshouse trial 2011; MN-2012= Minnesota field trial 2012

**Supplementary Table 2.** Combined analysis of variance (ANOVA) for grain Zn concentration at St Paul 2011glasshouse and 2012 field data

| Source of variation | DF | SS | MS | VR | Fpr | %SST |
| --- | --- | --- | --- | --- | --- | --- |
| Genotype (G) | 295 | 75423 | 256 | 6 | < 0.001 | 32 |
| Location (L) | 1 | 83954 | 83954 | 1982 | < 0.001 | 36 |
| Interaction (G x L, GEI) | 295 | 50139 | 170 | 4 | < 0.001 | 21 |
| Replication (R) | 1 | 0 | 0 | 0 | 1 | 0 |
| Residual | 591 | 25040 | 43 | - | - | 11 |
| Total | 1183 | 234555 | - | - | - | 100 |

GEI = genotype-by-environment/location interaction; Rep = replication; DF = degree of freedom; SS = sum of squares; MS = mean of squares; VR = variance ratio; Fpr = Fprobability statistics; %SST = percentage of total sum of square

**Supplementary Table 3**. HvZIFL genes identified using BLAST and associated 50K SNP markers with chromosome location and position on Morex v3 reference genome, with Minimum Allele Frequency and LOD score for grain zinc concentration shown.

| HvZIF genes SNP markers | 50K SNP marker | Chr | Position (bp) | LOD | *MAF |
| --- | --- | --- | --- | --- | --- |
| HORVU.MOREX.r3.1HG0003620 | JHI-Hv50k-2016-7793 | 1H | 6995151 | 4.56 | 0.154 |
| HORVU.MOREX.r3.1HG0003620 | JHI-Hv50k-2016-7841 | 1H | 7004595 | 4.56 | 0.027 |
| HORVU.MOREX.r3.1HG0003620 | JHI-Hv50k-2016-7843 | 1H | 7004854 | 4.56 | 0.058 |
| HORVU.MOREX.r3.1HG0047250 | JHI-Hv50k-2016-23511 | 1H | 309843016 | 4.56 | 0.110 |
| HORVU.MOREX.r3.1HG0050780 | JHI-Hv50k-2016-26139 | 1H | 338484237 | 4.62 | 0.144 |
| HORVU.MOREX.r3.1HG0083900 | JHI-Hv50k-2016-49079 | 1H | 531633198 | 4.56 | 0.000 |
| HORVU.MOREX.r3.2HG0183300 | JHI-Hv50k-2016-105924 | 2H | 565351361 | 4.57 | 0.079 |
| HORVU.MOREX.r3.2HG0171550 | JHI-Hv50k-2016-98791 | 2H | 567328834 | 4.56 | 0.099 |
| HORVU.MOREX.r3.3HG0255820 | JHI-Hv50k-2016-169537 | 3H | 191823532 | 4.56 | 0.072 |
| HORVU.MOREX.r3.3HG0255820 | JHI-Hv50k-2016-169533 | 3H | 191827763 | 4.56 | 0.434 |
| HORVU.MOREX.r3.3HG0260800 | JHI-Hv50k-2016-172407 | 3H | 248154452 | 4.56 | 0.443 |
| HORVU.MOREX.r3.3HG0329110 | JHI-Hv50k-2016-224589 | 3H | 693669003 | 4.56 | 0.147 |
| HORVU.MOREX.r3.3HG0329110 | JHI-Hv50k-2016-224622 | 3H | 693682387 | 4.56 | 0.141 |
| HORVU.MOREX.r3.4HG0340330 | JHI-Hv50k-2016-233527 | 4H | 29599114 | 4.69 | 0.110 |
| HORVU.MOREX.r3.4HG0356550 | JHI-Hv50k-2016-240342 | 4H | 148185634 | NA | 0.021 |
| HORVU.MOREX.r3.4HG0395230 | SCRI_RS_197889 | 4H | 525428416 | 4.60 | 0.476 |
| HORVU.MOREX.r3.4HG0416570 | JHI-Hv50k-2016-273928 | 4H | 604961366 | 4.56 | 0.075 |
| HORVU.MOREX.r3.4HG0416570 | JHI-Hv50k-2016-273925 | 4H | 604961732 | 4.57 | 0.072 |
| HORVU.MOREX.r3.4HG0416570 | JHI-Hv50k-2016-273924 | 4H | 604961735 | 4.57 | 0.072 |
| HORVU.MOREX.r3.4HG0416570 | JHI-Hv50k-2016-273923 | 4H | 604962083 | 4.56 | 0.055 |
| HORVU.MOREX.r3.4HG0409580 | JHI-Hv50k-2016-265265 | 4H | 619699160 | 4.61 | 0.188 |
| HORVU.MOREX.r3.5HG0462190 | JHI-Hv50k-2016-298545 | 5H | 345074509 | 4.86 | 0.120 |
| HORVU.MOREX.r3.5HG0476390 | JHI-Hv50k-2016-305116 | 5H | 406868558 | 4.56 | 0.199 |
| HORVU.MOREX.r3.5HG0476220 | JHI-Hv50k-2016-305093 | 5H | 453611774 | 4.56 | 0.161 |
| HORVU.MOREX.r3.6HG0543400 | JHI-Hv50k-2016-373984 | 6H | 12380529 | 4.56 | 0.295 |
| HORVU.MOREX.r3.6HG0543410 | JHI-Hv50k-2016-374002 | 6H | 12893653 | NA | 0.000 |
| HORVU.MOREX.r3.6HG0543410 | JHI-Hv50k-2016-374006 | 6H | 12893773 | 4.56 | 0.075 |
| HORVU.MOREX.r3.6HG0543590 | JHI-Hv50k-2016-374232 | 6H | 13076863 | 4.56 | 0.336 |
| HORVU.MOREX.r3.7HG0729020 | JHI-Hv50k-2016-494113 | 7H | 598439150 | 4.56 | 0.116 |

*Minor Allele Frequency; Chr =chromosome; bp = distance in base pairs; LOD = logarithm of ODDs (-log_10_P where P= significance probability level)

**Supplementary Table 4**. Summary of QTLs associated with grain Zn concentration in glasshouse trial, field trial and combined data at St Paul, Minnesota in 2011 and 2012 using the BLINK model

| QTL | QTL interval on Morex v3 | The lead marker position | Marker name | Trial | LOD | D* (Mb) | Potential candidate |
| --- | --- | --- | --- | --- | --- | --- | --- |
| Qzn1-2H | 2H_189225 2H_25132586 | 2H_ 8931234 | JHI-Hv50k- 2016-63311 | 2011 | 8.2 | 2.7; 7.9 | PCR1; PCR2 |
| Qzn2-2H | 2H_565415571 2H_594979690 | 2H_565791463 | JHI-Hv50k- 2016-106141 | 2012 | 7.7 | 9.7; 5.7; 6.5 | YSL2; YSL3; VIT2 |
| Qzn3-4H | 4H_490994 4H_14283002 | 4H_ 6501884 | JHI-Hv50k- 2016-228347 | 2011 | 5.9 | 0.30 | ZIP7 |
| Qzn4-4H | 4H_586552771 4H_589622088 | 4H_586552947 | JHI-Hv50k- 2016-265280 | 2011; 2012; Both* | 13.0 | 0 | ZIFL1 |
| Qzn5-6H | 6H_529161991 6H_556944786 | 6H_547245788 | JHI-Hv50k- 2016-426471 | 2011 | 6.9 | 7.7 | HMA |

QTL= quantitative trait loci, QTL interval= boundary of candidate genes within the specified genome region set by the linkage disequilibrium analysis; LOD = limit of detection, D*= distance in mega-base pairs of the candidate gene from the significantly associated marker

**Supplementary Table 5**. Primer sequences to amplify the whole ZIFL1 gene designed based on Morex version 1 (HORVU4Hr1G081570.1)

| No. | Forward primers (5’ to 3’) | Reverse primers (5’ to 3’) |
| --- | --- | --- |
| 1 | ACAGCACCAGCTAGCAATGG | ATTGCCAGAAACATGGCCTA |
| 2 | TCAGGATGCAGAGACATGGA | GAAAAGGATGGGCACTATGG |
| 3 | TGTGGTGCGTCAAAATATGC | GCATGCCCCTTAATTGTTTT |
| 4 | CCATGGAAACGCCATAACAT | GACAAGAATTTTGGAACGGAGT |
| 5 | GGTGTGTATGCCGGTCTTCT | GGCAAGCATGCCATTTAGAG |
| **6** | **TGGCTATTGCCACTAGAATGC** | **CCTGCGCTAAGTATCCTCCA** |
| 7 | TGGGGCATAGGTCTTGTTGT | TGTTGCCTTTTCAGGTTTGA |
| 8 | GGTTTCACACATTTAAAGCTAAACA | AAGACATCAATGGCCAGTTCTTA |
| **9** | **TCCCCATAGAGAAGTTCGACA** | **TCAGTGTGGGTCGAAAAGTG** |
| 10 | AAACAGTCCAAAGGGAACCA | CACAGCTGCAATACGAGATGA |
| 11 | TGCAACGGTGTCATTCTTTC | GGCCCATGAGAACCTACATT |
| 12 | AAGCCTACCAACACCATCTTT | TCCTCCGTATGGGAAAAAGT |

Primer sets in bold are markers for the INDEL and the diagnostic SNP: Primer set 6 amplifies the INDEL region while Primer set 9 amplifies the diagnostic marker

**Supplementary Table 6.** KASP primers designed to amplify the diagnostic SNP marker (JHI-50k-2016-265280)

| No | Primer | Primer sequences (5’ to 3’) |
| --- | --- | --- |
| 1 | VIC primer | GAAGGTCGGAGTCAACGGATTCTTTTCATCTAAAGATGTCGGCCAC |
| 2 | FAM primer | GAAGGTGACCAAGTTCATGCTTTTCATCTAAAGATGTCGGCCAA |
| 3 | Common primer | GTTTGTAAYTTATACCTGAAGCTGCAAGAA |

**Supplementary Table 7**. Haplotype sequences of the European Barley and Wheat Legacy collection (WHEALBI) from exome-captured data

| Haplotype* | Sequences of 80 SNP markers in HvZIFL1 | Frequency |
| --- | --- | --- |
| Hap_1 | GACCGCCCTGGCCAGAAAGCGGCGGATCCAGCTGCGATTGTCCTCTGTACACGACTGTACCGCATCGGCTTGGGCCTCTT | 4 (0.0093) |
| Hap_2 | GACCGCCCTGGCCAGAAAGCGGCGGATCCAGCTGTGATTGTCCTCTGTACACGACTGTACCGCATCGGCTTGGGCCTCTT | 6 (0.0139) |
| Hap_3 | GACTACCACGGTCCGAAAGAGTCGAGTCCAACTATAATCGTCCTCTGCACACCGCTGTGACGCCTCGGCCTAGGCCTCCT | 1 (0.0023) |
| Hap_4 | GACTACCCCGGTCCGAAAGAGTCGAGTCCAACTATAATCGTCCTCTGCACACCGCTGTGACGCCTCGGCCCAGGCCTCCT | 2 (0.0509) |
| Hap_5 | GACTACCCCGGTCCGAAAGAGTCGAGTCCAACTATAATCGTCCTCTGCACACCGCTGTGACGCCTCGGCCTAGGCCTCCT | 5 (0.0116) |
| Hap_6 | GACTACCTCGGTTCGCAAGCGTCGAGTCCAACTATAATTGTTCTCTGTACACCGCTGTGACGCCTCGGCTTAGGCTTGTT | 1 (0.0023) |
| Hap_7 | GACTACCCCGGTCCGAAAGCGTCGAGTCCAACTACAATTGTCCTCGGTACGCCGCTGTGACGCCTTGGCTTAAGGTTGTT | 1 (0.0023) |
| Hap_8 | GACTAGCCCGGTCCGAAAGCGTCGAGTCCAACTATAATTGTCCTCTGCACAGCGCTGTGACGCCTCGGCTTAGGCCTCTT | 1 (0.0023) |
| Hap_9 | GACTAGCCCGGTCCGAAAGCGTCGAGTCCAACTATAATTGTCCTCTGCGCAGCGCTGTGACGCCTCGGCTTAGGCCCCTT | 1 (0.0023) |
| Hap_10 | GACCGCACTGGCCCGAAAGCGGCGGATCCAGCTGTGATTGCCCTCTGTACACGACTGTACCGCCTCGGCTTGGACCTCTT | 1 (0.0023) |
| Hap_11 | GACCGCACTGGCCCGAAAGCGGCGGATCCAGCTGTGATTGTCCTCTGTACACGACTGTACCGCCTCGGCTTGGACCTCTT | 1 (0.0023) |
| Hap_12 | AACCACCCTGGCCCGAAAGCGGCGGATCCAGCTGTGATTGTCCTCTGTACACGACTGCGCCGCCTCGGCTTAGGCCTCTT | 1 (0.0023) |
| Hap_13 | GACCACCCCAGCCCGAACGCGGCAGATCCAGCTGTGATTGTCTTCTGTACACGACTATGCCGCCTCGACTTAGGCCTCTT | 1 (0.0023) |
| Hap_14 | GACCACCCTGGCCCGAAAGCGGCGGATCAAGCTGTGATTGTCCTCTGTACACGACCGTGCCGCCTCGGCTTAGGCCTCTT | 1 (0.0023) |
| Hap_15 | GACCACCCTGGCCCGAAAGCGGCGGATCCAGCTGTGATTGTCCTCTGTACACGACCGTGCCGCCTCGGCTTAGGCCTCTT | 35 (0.081) |
| Hap_16 | GACCACCCTGGCCCGAAAGCGGCGGATCCAGTTGTGATTGTCCTCTGTACACGACCGTGCCGCCTCGGCTTAGGCCTCTT | 1 (0.0023) |
| Hap_17 | AACCACCCTGGCCCGAAAGCGGCGGAACCTGCTGTGATTGTCCTCTCTACACGACTGTGCCGCCTCGGCTTGGGCCTCTT | 1 (0.0023) |
| Hap_18 | GACCACCCTGGCCCGAAAGCGGCGGATCCAGCTGTGATTGTCCGCTGTACACGACTGTGCCGCCTCGGCTTAGGCCTCTT | 20 (0.046) |
| Hap_19 | GACCACCCTGGCCCGAAATCGGCGGATCCAGCTGTGACTATCCTCTGTACACGACTGTGCCGCCTCGGCTTGGGCCTCTT | 12 (0.028) |
| Hap_20 | GACCACCCTGACCCGAAAGCGGCGGATCCAGCTGTGATTGTCCTCTGTACACGACTGTGCCGCCTCGGTTTGGGCCTCTT | 1 (0.0023) |
| Hap_21 | GACCACCCTGGCCCGAAAGCGGCGGATCCAGCCGTGATTGTCCTCTGTACACGACTGTGCCGCCTCGGCTTAGGCCTCTT | 1 (0.0023) |
| Hap_22 | GACCACCCTGGCCCGAAAGCGGCGGATCCAGCTGTGATTGTCCTCTGTACACGACTGTGCCGCCTCGGCTTAGGCCTCTT | 49 (0.113) |
| Hap_23 | GATCACCCTGGCCCGAAAGCGGCGGATCCAGCTGTGATTGTCCTCTGTACACGACTGTGCCGCCTCGGCTTAGGCCTCTT | 4 (0.0093) |
| Hap_24 | GACCACCCTGGCCCGAAAGCGGCGGATCCAGCTGTGATTGTCCTCTGTACACGACTGTGCCGCCTCGGTTTGGGCCTCTT | 212 (0.49) |
| Hap_25 | GACCACCCTGGCCCGAGAGCGGCGGATCCAGCTGTGATTGTCCTCTGTACACGACTGTGCCGCCTCGGCTTAGGCCTCTT | 1 (0.0023) |
| Hap_26 | GACCACCCTGGCCCGAAAGCGGTGGATCCAGCTGTGATTGTCCTCTGTACACGACTGTGCCGCCTCGGCTTAGGCCTCTT | 2 (0.0509) |
| Hap_27 | AACCACCCTGGCCCGAAAGCAGCGGAACCTGCTGTGATTGTCCTCTGTACACGACTGTGCCGCCTCGGCTTGGGCCTCTT | 2 (0.0509) |
| Hap_28 | AACCACCCTGGCCCGAAAGCGGCGGAACCTGCTGTGATTGTCCTCTGTACACGACTGTGCCGCCTCAGCTTGGGCCTCTT | 1 (0.0023) |
| Hap_29 | AACCACCCTGGCCCGAAAGCGGCGGAACCTGCTGTGATTGTCCTCTGTACACGACTGTGCCGCCTCGGCTTGGGCCTCTT | 36 (0.083) |
| Hap_30 | AACCACCCTGGCCCGAAAGCGGCGGAATCTGCTGTGATTGTCCTCTGTACACGACTGTGCCGCCTCGGCTTGGGCCTCTT | 1 (0.0023) |
| Hap_31 | GACCACCCTGGCCCGAAAGCGGCGGATCCAGCTGTGGTTGTCCTCTGTACACGACTGTGCCGCCTCGGTTTGGGCCTCTT | 1 (0.0023) |
| Hap_32 | GACCACCCCAGCCCGAACGCGGCAGATCCAGCTGTGATTGTCTTCTGTACACGACTGTGCCGCCTCGACTTAGGCCTCTT | 4 (0.0093) |
| Hap_33 | GACCACCCCAGCCCGAACGCGGCAGATCCAGCTGTGATTGTCTTTTGTACACGACTGTGCCGCCTCGACTTAGGCCTCTT | 2 (0.0509) |
| Hap_34 | GACCACCCTGGCCCAAAAGCGGCGGATCCAGCTGTGATTGTCCTCTGTAAACGAGTGTGCCGCCGCGGCTTAGGCCTCTT | 1 (0.0023) |
| Hap_35 | GACTACCCCGGTCCGAAAGCGTCGAGTCCAACTATAATTGTCCTCTGCACACCGCTGTGATGCCTCGGCTTAGGCCTCTC | 2 (0.0509) |
| Hap_36 | GACCACCCTGGCCCGAAAGCGGCGGATCCAGCTGTGATTGTCCTCTGTACACGACTGTGCCTCCTCGGCTTAGGCCTCTT | 14 (0.032) |
| Hap_37 | GACCACCCTGGCCCGAAAGCGGCGGATCCAGCTGTGATCGTCCTCTGTACACGACTGTGCCGTCTCGGCTTAGGCCTCTT | 1 (0.0023) |
| Hap_38 | GCCCACCCTGGCCCGAAAGCGGCGGATCCAGCTGTGATTGTCCTCTGTACACGACTGTGCCGCCTCGGCTTAGGCCTCTT | 1 (0.0023) |
| Total | | 432 |

*Sequences used to construct haplotypes were derived from 80 SNPs within HvZIFL1 gene of the WHEALBI collection (N= 432) exome capture data (alleles at the diagnostic marker JHI-Hv50k-2016-265280 is highlighted: the wildtype ‘T’ on the reverse strand is highlighted in yellow; the mutant (favourable) allele ‘G’ in red)

**Supplementary Table** **8**. Number and Frequency of SNP and INDEL polymorphisms

| Polymorphism | | SP1 | SP2 | SP3 | SP4 |
| --- | --- | --- | --- | --- | --- |
| INDEL | Wildtype (deletion) | 211(0.99) | 17(0.40) | 16(0.73) | 1(0.05) |
|  | Mutant (insertion) | 2(0.01) | 25(0.60) | 6(0.27) | 19(0.95) |
| Marker | Wildtype (A) | 160(0.75) | 42(1.00) | 16(0.72) | 20(1.00) |
|  | Mutant (C) | 53(0.25) | 0(0.00) | 6(0.28) | 0(0.00) |

INDEL= insertion-deletion; SP1= sub-population 1; SP2= sub-population 2; sub-population 3; SP3= sub-population 4; figures in bracket indicate frequency of genotypes within the sub-population groups with their corresponding SNP and INDEL polymorphisms

**Supplementary Table** **9.** List and frequency of the EEBC lines having INDEL within the HvZIFL1

| Population | List of genotypes with the INDEL | Frequency |
| --- | --- | --- |
| SP1 | EEBC_022, EEBC_177 | 2 (0.038) |
| SP2 | EEBC_005, EEBC_030, EEBC_060, EEBC_087, EEBC_114, EEBC_120, EEBC_147, EEBC_195, EEBC_213, EEBC_221, EEBC_223, EEBC_228, EEBC_232, EEBC_252, EEBC_253, EEBC_256, EEBC_258, EEBC_264, EEBC_271, EEBC_273, EEBC_274, EEBC_279, EEBC_281, EEBC_288, EEBC_293 | 25 (0.48) |
| SP3 | EEBC_058, EEBC_109, EEBC_227, EEBC_229, EEBC_233, EEBC_254 | 6 (0.12) |
| SP4 | EEBC_101, EEBC_102, EEBC_106, EEBC_225, EEBC_241, EEBC_245, EEBC_246, EEBC_250, EEBC_251, EEBC_255, EEBC_262, EEBC_275, EEBC_276, EEBC_277, EEBC_278, EEBC_280, EEBC_282, EEBC_285, EEBC_289 | 19 (0.37) |
| Total | | 52 (1.00) |

**Supplementary Table 10**. List and frequency of the EEBC lines having the favourable allele at the diagnostic marker JHI-Hv50k-2016-265280

| Population | List of genotypes with the favourable “C” allele | Frequency |
| --- | --- | --- |
| SP1 | EEBC_003, EEBC_009, EEBC_012, EEBC_015, EEBC_028, EEBC_031, EEBC_035, EEBC_036, EEBC_040, EEBC_044, EEBC_045, EEBC_046, EEBC_051, EEBC_056, EEBC_061, EEBC_068, EEBC_069, EEBC_072, EEBC_083, EEBC_085, EEBC_090, EEBC_115, EEBC_116 EEBC_118, EEBC_119, EEBC_123, EEBC_125, EEBC_137, EEBC_138, EEBC_142, EEBC_143, EEBC_146, EEBC_151, EEBC_152, EEBC_158, EEBC_159, EEBC_165, EEBC_172, EEBC_178, EEBC_179, EEBC_186, EEBC_200, EEBC_202, EEBC_203, EEBC_208, EEBC_210, EEBC_215, EEBC_216, EEBC_219, EEBC_222, EEBC_226, EEBC_247, EEBC_259 | 53 (0.25) |
| SP3 | EEBC_157, EEBC_191, EEBC_194, EEBC_237, EEBC_257, EEBC_270 | 6 (0.27) |
| Total | | 59 (1.00) |

# Supplementary Figures

1. B)

**Supplementary Figure 1.** A) Population structure of the world barley collection in Darrier et al., (2019) combined with the EEBC panel. The combined analysis reveals four distinct clusters of 2-row spring (top, black), 2-row mixed spring and winter types (bottom left, blue), 6-row Asian (bottom right, red); B) Molecular variance within and between populations.

A)

B)

**Supplementary Figure 2**. Comparison of GWAS models (pKWmEB, BLINK and MLM from top to bottom) using trials data from: A) glasshouse; B) field in Saint Paul, Minnesota.


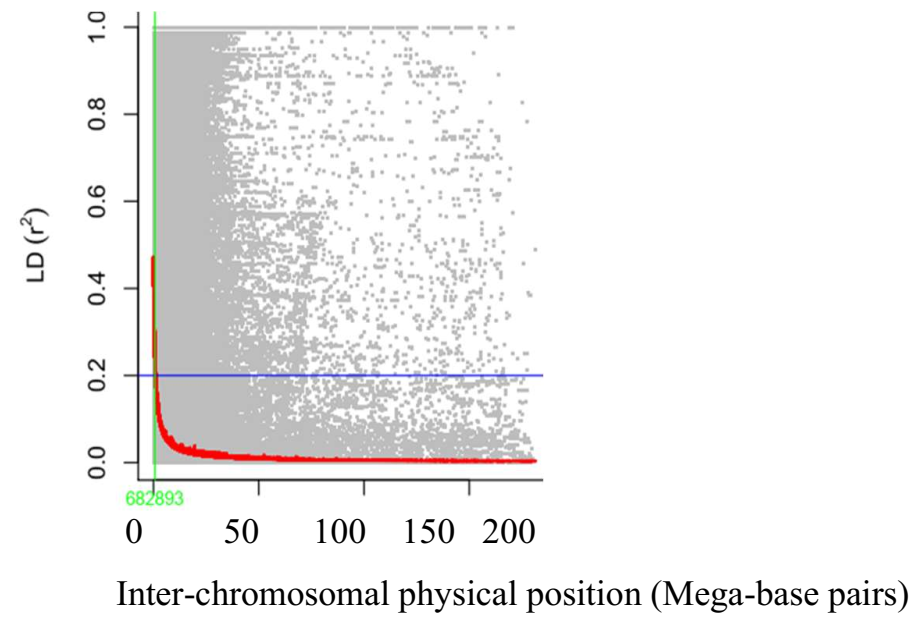


**Supplementary Figure 3.** Inter-chromosomal linkage disequilibrium (LD) decay of the EEBC germplasm. X-axis shows LD in mega-base pairs (Mbps); Y-axis measures LD in square allele frequency (r^2^); blue horizontal line indicates LD significance cutoff; green vertical line indicates minimum distance (base-pairs) within which LD decays.


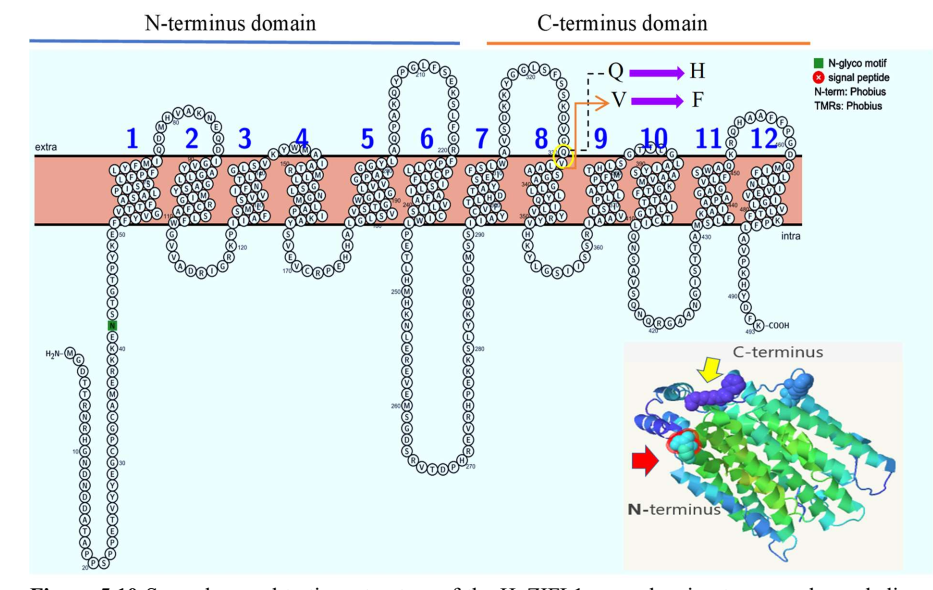


**Supplementary Figure 4**. Secondary and tertiary structure of the HvZIFL1 gene showing transmembrane helices. Each transmembrane helix (TMH) is numbered from 1 to 12. Histidine replaces Glutamine at the entrance of TMH8 and Phenylalanine replaces the adjacent Valine.


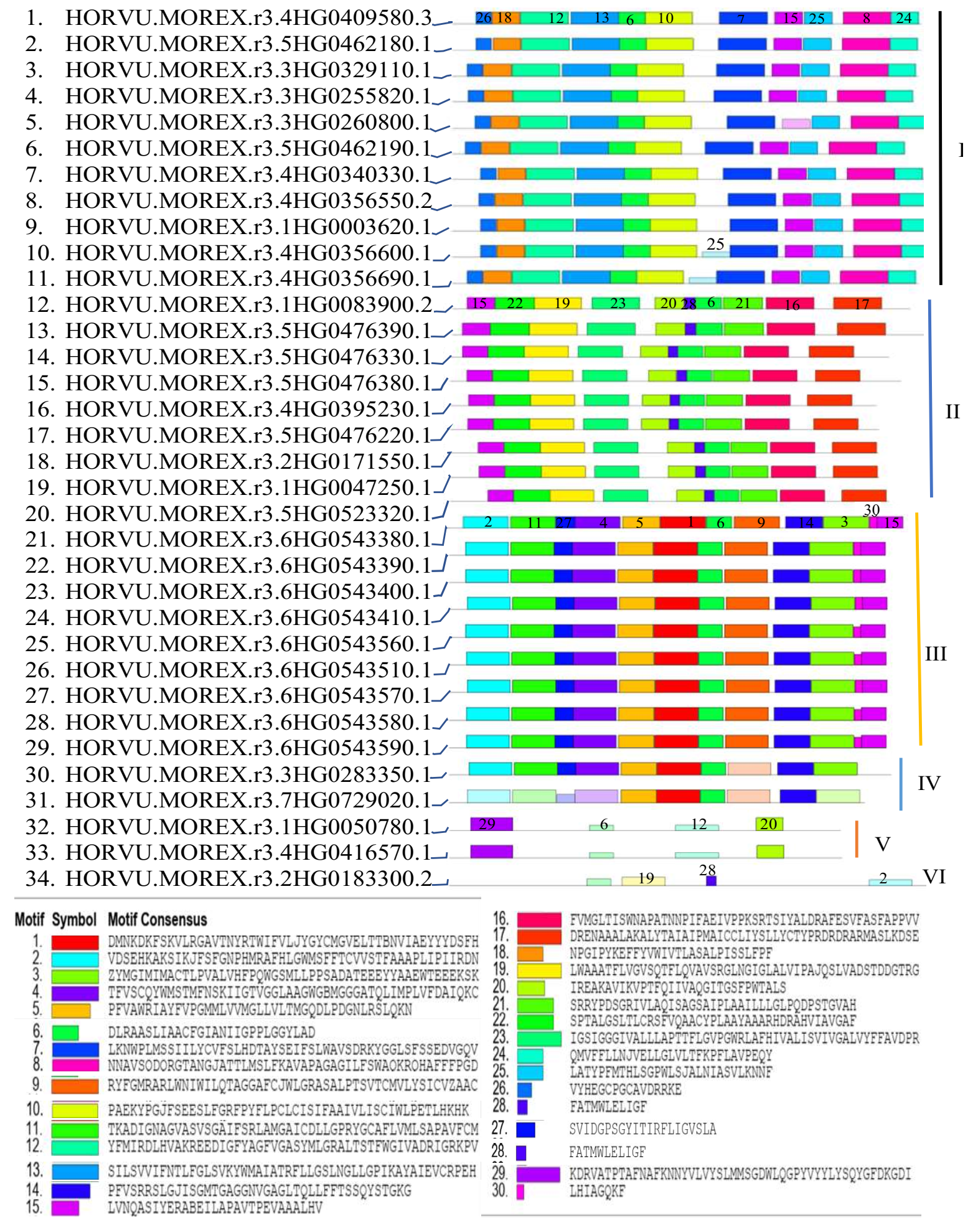


**Supplementary Figure 5.** Protein motifs of HvZIFL1 genes of the barley genome.


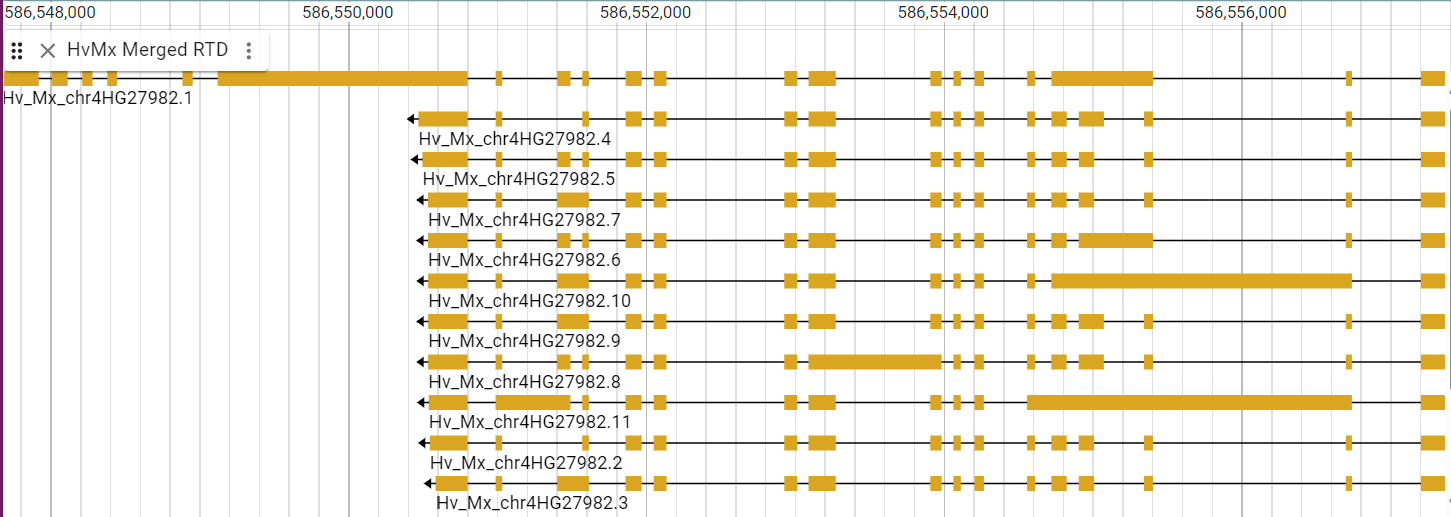


**Supplementary Figure 6**. HvZIFL1 gene (Hv_Mx_chr4HG27982) alternate transcripts of the cv. Morex taken from [[Morex Gene Atlas - Hv_Mx_chr4HG27982 (hutton.ac.uk)](https://ics.hutton.ac.uk/morexgeneatlas/gene.cgi?seq_name=Hv_Mx_chr4HG27982%20&dataset=HvMxRTD)(Guo et al., 2025)]. Transcript 5 is the most annotated in previous versions of Morex reference genome assemblies.

A) B) C) D) E) F)

**Supplementary Figure 7**. ZIFL1 gene expression in transcripts per million (TPM) on the Y-axis and tissue samples with treatment conditions on the X-axis extracted from Morex Gene atlas [[Morex Gene Atlas - Hv_Mx_chr4HG27982 (hutton.ac.uk)](https://ics.hutton.ac.uk/morexgeneatlas/gene.cgi?seq_name=Hv_Mx_chr4HG27982&dataset=HvMxRTD)(Guo et al., 2025)]. A) 11 alternative transcripts of ZIFL1 gene; B) 7 days old roots with Cadmium (Cd), Zinc (Zn), and Copper (Cu) treatments in three consecutive replications, from left to right: Cd control >> Cd treated >> Cu control >> Cu treated >> Zn control >> Zn treated; C) similar sequence of treatments for 7 days old shoots; D) 32 days old roots in NaCl treatments in three consecutive replications from left to right: NaCl control >> 150 mM NaCl treatment >> 300 mM NaCl treatment; E) similar treatment sequence for 32 days old shoots; F) 2-3 leaf stage roots with Abscisic Acid (ABA) treatment in three consecutive replication from left to right: 1 hour (1hr) ABA control >> 3hr ABA control >> 6hr ABA control >> 24hr ABA control >> 1hr ABA treated >> 3hr ABA treated >> 6hr ABA treated >> 24 hr ABA treated.
